# Prescription of Silexan Is Associated with Less Frequent General Practitioner Repeat Consultations Due to Disturbed Sleep Compared to Benzodiazepine Receptor Agonists: A Retrospective Database Analysis

**DOI:** 10.3390/healthcare11010077

**Published:** 2022-12-27

**Authors:** Tillmann Krüger, Eva-Maria Becker, Karel Kostev

**Affiliations:** 1 Department of Psychiatry, Social Psychiatry and Psychotherapy, Division of Clinical Psychology & Sexual Medicine, Hanover Medical School, 30625 Hanover, Germany; 2 Center for Systems Neuroscience, 30559 Hanover, Germany; 3 Epidemiology, IQVIA Unterschweinstiege 2–14, 60549 Frankfurt am Main, Germany

**Keywords:** retrospective study, Silexan, subsyndromal generalized anxiety disorder, sleep disorder, Z-drugs

## Abstract

The aim of the present study was to analyze the association between the prescription of Silexan and the recurrence of general practitioner (GP) repeat consultations because of disturbed sleep versus benzodiazepine receptor agonists including zolpidem, zopiclone, and zaleplon (Z-drugs). This retrospective cohort study was based on data from the IQVIA Disease Analyzer (DA) database. The study included adult patients treated by 1284 GPs in Germany with a documented sleep disorder and their first prescription of Silexan or Z-drug (prescription between January 2010 and October 2020). The recurrence of seeking medical advice because of sleep disorders in the 15–365 days after the first prescription was evaluated. Multivariate regression models were used, adjusted for age, sex, insurance status, and defined co-diagnoses. Data were available for 95,320 (Silexan: 5204; Z-Drug: 90,526) patients. In total, 15.6% of the Silexan patients and 28.6% of the Z-drug patients had a further documented GP consultation because of a sleep disorder. Silexan prescription was associated with significantly lower odds of recurrent sleep disorder diagnosis in the 15–365 days after the index date (Odds Ratio (OR): 0.56; 95% confidence intervals (CI): 0.51–0.60), although mental burden levels appeared higher in this group. Our study shows that the prescription of Silexan to adult patients consulting GPs for disturbed sleep results in less frequent repeat consultations than Z-drugs. This may support Silexan’s role as an efficacious, self-enabling, well-tolerated, and sustained treatment option. Because Silexan is a proven anxiolytic, its impact in improving undiagnosed anxiety disorders may have had a lasting effect for certain patients.

## 1. Introduction

Sleep disorders, which are defined as conditions disturbing normal sleep patterns, are one of the most common diagnoses in clinical practice [1]. They include insomnia, parasomnia, central disorders of hypersomnolence, sleep-related breathing disorders, and others [2]. Insomnia affects approximately 6% of the population in Western industrialized countries, with a lifetime prevalence of parasomnia of between 4 and 67% depending on the definition shown in a large population-based study from Denmark [3]. In Germany, the prevalence of insomnia was reported to be 5.7% [4].

Sleep disorders are coded as G47 and F51 in the International Classification of Diseases (ICD-10). This code applies not only to full syndromal disorders as defined in diagnostic systems such as the DSM-V or ICD-11 but also to subsyndromal disorders that are frequently seen in the general population. Volz et al. recently proposed criteria for subsyndromal GAD that stipulate sleep problems as one of six core symptoms [5]. Anxiety disorders are an important risk factor for impaired sleep in the general population [6]. Therefore, many patients requesting treatment for insomnia may suffer from underlying anxiety [7].

German guidelines for the treatment of sleep disorders [8] recommend behavioral therapy for insomnia prior to starting drug treatment. Short-term drug therapy (up to 4 weeks) with benzodiazepines and benzodiazepine receptor agonists, also known as Z-drugs, has shown a strong efficacy in insomnia patients in the form of positive changes in both subjective and polysomnographic sleep parameters. Z-drugs contain zolpidem, zopiclone, and zaleplon. The mechanisms of action of these drugs have already been described elsewhere [9,10]. Previous research has reported an association between one of these drugs and an increased risk of suicide [11], fractures [12], and injuries [13].

Guidelines also mention phytopharmaceutical drugs for sleep disorders. A number of meta-analyses have been carried out on the topic of phytotherapy for insomnia [14,15,16], which show a slight superiority of valerian over placebo. However, the European Medical Agency (EMA) has approved a recommendation for the use of valerian in the treatment of sleep disorders based on its well-established use. Silexan is an active substance with an essential oil produced from *Lavandula angustifolia* flowers (Silexan^®^ is a proprietary lavender oil of Dr. Willmar Schwabe GmbH & Co.KG, Karlsruhe, Germany) that complies with and exceeds the European Pharmacopoeia quality definition for the monograph lavender oil. Several studies have shown positive effects of Silexan on symptoms of subsyndromal anxiety or GAD compared to conventional treatment or placebo groups [17,18,19]. Using path analysis, Seifritz et al. demonstrated that Silexan exerts a secondary sleep-improving effect for patients with anxiety disorders [20]. 

While benzodiazepine receptor agonists such as Zolpidem and Zopiclone only provide symptomatic relief of insomnia, an anxiolytic drug such as Silexan can be expected to provide more effective treatment where sleep problems arise from an underlying anxiety disorder. This difference should translate into a declining need for insomnia treatment over time with Silexan as compared to Z-drugs. As sleep disorders have a high prevalence and may negatively impact quality of life as well as both somatic and mental health, investigations of the effectiveness of sleep disorder therapy are of high clinical relevance.

Hence, the aim of the present study is to analyze the association between the prescription of Silexan and the recurrence of GP consultations for sleep disorders within one year in comparison to treatment with Z-drugs.

## 2. Methods

### 2.1. Database

This analysis was performed using the IQVIA Disease Analyzer (DA) database, which contains case-based information provided by office-based physicians (both GPs and specialists) in Germany. Information is available on patient demographics, drug prescriptions, concomitant medication, comorbid conditions, sick leave, and referrals to hospitals. Nearly 3000 office-based physicians, representing approximately 3% of all German practices, provide information directly and in an anonymous format from the computer systems used in their practices (DA status date: March 2022). Practices can be categorized into 10 classes according to the physician’s specialty (GPs and various specialists). The sample of practices included is representative of the overall situation in Germany with regard to geography, covering eight major German regions [21]. 

The database has already been proven suitable for pharmacoepidemiological and pharmacoeconomic studies [21]. IQVIA ensures the accuracy, consistency, and completeness of the data. Evident data limitations are communicated immediately by IQVIA, allowing the analysis to be adapted accordingly.

### 2.2. Study Population and Outcome

**Figure 1:**
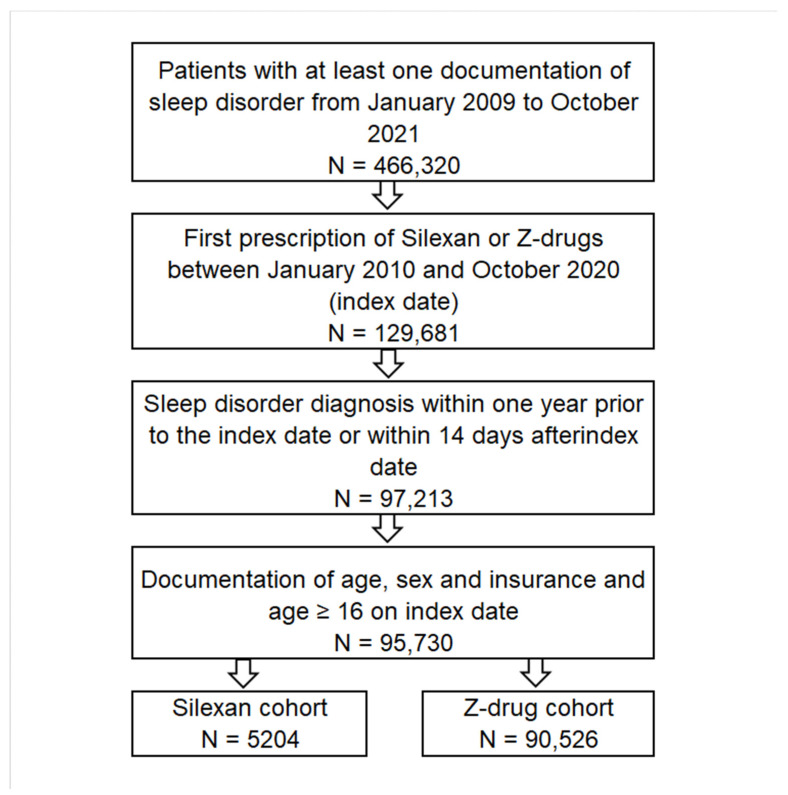
Selection of study patients.

This study included adult patients treated in general practices (GPs) in Germany who received their first Silexan or Z-drug prescription between January 2010 and October 2020 (the index date) and were diagnosed with a sleep disorder (ICD-10: G47.0 or G47.9) within one year before or within 14 days after the index date. Patients were excluded if both Silexan and Z-drugs were prescribed at the same time (Figure 1). The main outcome of the study was the proportion of patients treated with Silexan versus Z-drugs with repeated documentation of sleep disorder diagnoses 15–365 days after the index date.

### 2.3. Statistical Analyses

Baseline characteristics were compared between the Silexan vs. Z-Drug cohorts using the Wilcoxon signed-rank test or the chi-square test. The normality of the age data distribution was evaluated using the Kolmogorov–Smirnov test. The time to the first repeat GP consultation for sleep disorder was shown using Kaplan–Meier curves.

Multivariable logistic regression models adjusted by age, sex, insurance status, and defined co-diagnoses (including depression (ICD-10: F32 and F33), anxiety disorder (ICD-10: F41), reaction to severe stress and adjustment disorder (ICD-10: F43), diabetes mellitus (ICD-10: E10–E14), back pain (ICD-10: M54), osteoarthritis (ICD-10: M15–M19), chronic bronchitis or chronic obstructive lung disease (COPD) (ICD-10: J42–J44), and cancer (ICD-10: C00–C97)) were used to evaluate the association between the prescription of Silexan and a repeated documentation of a sleep disorder. Each of these disorders was diagnosed in more than 5% of the study patients and is known to be associated with sleep disorders. All of these variables were included in the model regardless of their significance in order to control for confounding.

Due to the age differences between the two cohorts, regression models were performed separately by age group (16–30, 31–40, 41–50, 51–60, 61–70, and >70 years), adjusted for age, sex, insurance status, and the co-diagnoses listed above.

A *p*-value of <0.05 was considered statistically significant. All analyses were performed using SAS 9.4 (SAS Institute, Cary, NC, USA).

The guidelines for reporting observational studies provided through the Strengthening the Reporting of Observational Studies in Epidemiology (STROBE) initiative were followed.

### 2.4. Ethical Statement

The database used includes only anonymized data in compliance with the regulations of the applicable data protection laws. German law allows the use of anonymous electronic medical records for research purposes under certain conditions. According to this legislation, it is not necessary to obtain informed consent from patients or approval from a medical ethics committee for this type of observational study that contains no directly identifiable data.

Because patients were only queried as aggregates and no protected health information was available for queries, no Institutional Review Board approval was required for the use of this database or the completion of this study.

## 3. Results

### 3.1. Basic Characteristics of the Study Sample

A total of 5204 Silexan patients and 90,526 Z-drug patients were included in the study. Table 1 shows the baseline characteristics of the study patients. Patients receiving Silexan were younger (mean 48.7 vs. 61.5 years) and more often female (66.4% vs. 59.6%). The proportions of patients with depression (21.7% vs. 18.4%), anxiety disorder (11.3% vs. 5.5%), and reaction to severe stress and adjustment disorder (13.2 vs. 8.0%) were higher in those receiving Silexan than in those treated with Z-drugs. By contrast, somatic disorders such as diabetes mellitus, osteoarthritis, chronic bronchitis/COPD, and cancer were found more often among patients with Z-drug prescriptions than in those taking Silexan.

### 3.2. New Sleep Disorder Diagnoses

**Figure 2:**
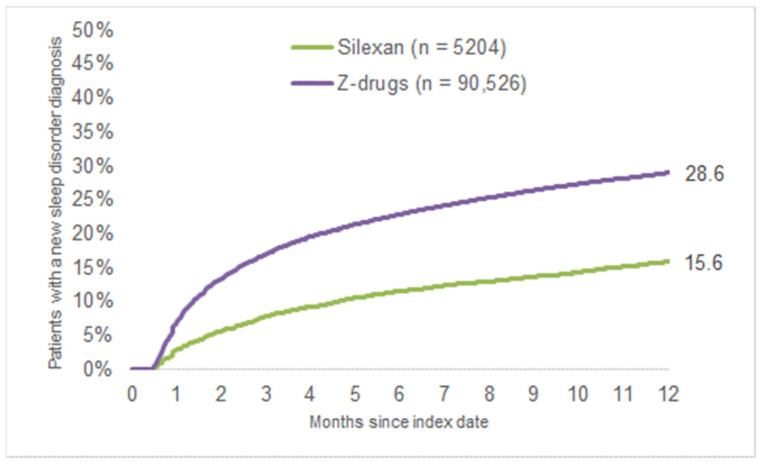
Kaplan–Meier curves for the time to the first repeat GP consultation for disturbed sleep in patients treated with Silexan versus Z-drugs.

**Figure 3:**
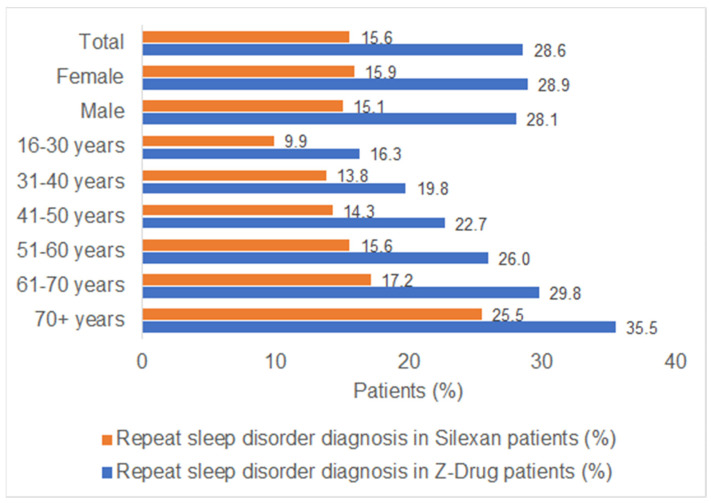
Proportion of Silexan and Z-drug patients with a repeat diagnosis of sleep disorder within 15 days up to one year after the index date.

At least one new sleep disorder diagnosis was documented within 15–365 days after the index date for 15.6% of the Silexan patients and 28.6% of the Z-drug patients (Figure 2). Furthermore, Figure 3 shows a difference in the proportion of patients with repeatedly documented diagnoses separated by age group: just 9.9% of the Silexan patients aged between 16–30 years had a further documented sleep disorder diagnosis, whereas 25.5% of the Silexan patients aged over 70 years received a repeat diagnosis.

The results of the multivariate logistic regression model are displayed in Table 2 and show that a Silexan prescription was associated with significantly lower odds of a repeat sleep disorder diagnosis within a period of 15–365 days after the index date (OR: 0.56; 95% CI: 0.51–0.60). Furthermore, higher age, depression, anxiety disorder, diabetes, back pain, osteoarthritis, chronic bronchitis or COPD, and cancer were also significantly and positively associated with the new sleep disorder diagnosis. 

Table 3 shows the effect of Silexan vs. Z-drugs in the regression models stratified by age group. The significant effect of Silexan on the reduced odds of new sleep disorders was similar among all age groups.

## 4. Discussion

### 4.1. Main Results

This retrospective study, based on a large sample of around 95,000 patients consulting a GP for disturbed sleep in Germany, resulted in the finding that the prescription of Silexan was significantly associated with a lower likelihood of repeat consultation because of a sleep disorder within a period of 15 to 365 days after prescription compared to Z-drugs. These significant associations were observable within all age groups, with an odds ratio of between 0.48 and 0.65. The present study was based on a large population of individuals, both women and men, across all age groups, using longitudinal data. 

### 4.2. Interpretation of Results

Previous studies have established the efficacy of Silexan compared to placebo for the treatment of subthreshold anxiety and generalized anxiety disorder [19]. Significant improvement in anxiety-related symptoms such as depressive symptoms, physical symptoms, and disturbed sleep has been demonstrated in these trials [20,22,23]. Substantial anxiolytic effects compared to placebo developed over the first 2 treatment weeks, and the treatment effect became more pronounced with continued treatment. By contrast, significant improvements in sleep quality have been observed after 6 weeks of treatment or more. By means of mediation analysis, Seifritz et al. demonstrated that Silexan exerts a secondary sleep-improving effect almost exclusively through its anxiolytic action rather than by sedation [20]. 

Castro et al. analyzed the effect of Zolpidem in oral and sublingual form for adults in a 3 month randomized trial [24]. Medication was taken by study participants “as needed”. The number of doses taken as well as total sleep time, sleep-onset latency, and sleep efficiency remained relatively stable over 13 weeks of treatment. Comparable results were reported by Walsh et al. [25].

The present study compared the effects of Silexan and those of Z-drugs for the first time by analyzing recurring GP consultations because of disturbed sleep. 

### 4.3. Study Limitations

The present study is subject to a number of limitations that need to be addressed. The first of these is that the severity of the sleep disorder is not specified in the database and could not be analyzed in the present study. It is possible that Z-drug patients had more severe symptoms and a higher degree of sleep disorders, meaning a renewed documentation of sleep diagnosis appears more frequently for them. 

Second, patients taking Silexan may seek repeated consultations less frequently because, in contrast to those treated with Z-drugs, they do not require a prescription to obtain the drug from a pharmacy as it is an OTC (over the counter) drug.

Third, the prescribed dosage of study drugs for each patient was not analyzed and may bias the study results. However, this difficulty exists only with Z-drug prescriptions, since in the case of Silexan, patients usually take one soft capsule (corresponding to 80 mg of lavender oil per day).

Fourth, patients receiving Silexan had a comorbid depression or anxiety disorder significantly more often. This indicates that Silexan might have been prescribed more frequently to patients with impaired sleep secondary to psychiatric comorbidity instead of pure insomnia. However, we observed a group-specific difference with an OR of around 0.5 even after controlling for co-morbid anxiety disorders, reactions to severe stress, adjustment disorders, and depression. The ICD-10 code F41 we used to identify co-morbid anxiety and comprises generalized anxiety disorder but also subsyndromal anxiety, i.e., mixed anxiety and depressive disorder, other mixed or specified anxiety disorders, and unspecified anxiety disorders. Nevertheless, syndromal and subsyndromal anxiety disorders are remarkably underdiagnosed in primary care settings and therefore may not have been captured in the database if the GP focused on treating the prominent symptom of disturbed sleep [26,27]. We hypothesize the longer-lasting treatment effect from the use of Silexan is due in part to Silexan’s clinically proven efficacy in the treatment of subsyndromal anxiety disorders and agitation, which could be the cause of the sleep disturbances. Especially in view of the very good tolerability and absence of dependence effects of Silexan compared to Z-drugs, the high level of acceptance of the drug Silexan among patients could lead them to cope independently with the stressors responsible for their sleep disorders. Further studies are necessary to analyze this effect in greater detail.

### 4.4. Clinical Implications

Disturbed sleep is among the core symptoms in patients with anxiety disorders. Anxiety disorders are an important risk factor for impaired sleep in the general population. As the current data show, a considerable proportion of patients requesting treatment for insomnia suffer from underlying anxiety or another mental burden. GPs are advised to screen for underlying anxiety in all patients presenting with the complaint of disturbed sleep. Two simple questions can be sufficient for this purpose, as used in the GAD-2 questionnaire (feeling nervous, anxious, or on edge and not being able to stop or control worrying over the last two weeks) [15]. Silexan may be a preferable treatment option for these patients compared to Z-drugs.

## 5. Conclusions

Our study provides evidence that the prescription of Silexan compared to Z-drugs to adult patients consulting their GPs for disturbed sleep resulted in less frequent repeat consultations. This further strengthens Silexan’s profile as a well-accepted, self-enabling, and efficacious treatment option. Because Silexan is a proven anxiolytic, its impact in improving undiagnosed anxiety disorders may have had a lasting effect for certain patients.

## Figures and Tables

**Table 1:** Basic characteristics of the study sample after propensity score matching.

Variable	Patients with Silexan Prescription	Patients with Z-Drug Prescription	*p*-Value
N	5204	90,526	
Age mean (SD)	48.7 (19.2)	61.5 (18.2)	<0.001
16–30 years [*n* (%)]	1172 (22.5%)	5948 (6.6%)	<0.001
31–40 years [*n* (%)]	680 (13.1%)	7255 (8.0%)	<0.001
41–50 years [*n* (%)]	938 (18.0%)	12,090 (13.4%)	<0.001
51–60 years [*n* (%)]	982 (18.9%)	16,669 (18.4%)	0.409
61–70 years [*n* (%)]	594 (11.4%)	14,668 (16.2%)	<0.001
>70 years [*n* (%)]	838 (16.1%)	33,896 (37.4%)	<0.001
Sex: female [*n* (%)]	3456 (66.4%)	53,982 (59.6%)	<0.001
Private health insurance [*n* (%)]	622 (12.0%)	9020 (10.0%)	<0.001
Depression	1128 (21.7%)	16,665 (18.4%)	<0.001
Anxiety disorder	586 (11,3%)	4945 (5.5%)	<0.001
Reaction to severe stress and adjustment disorder	685 (13.2%)	7226 (8.0%)	<0.001
Diabetes	345 (6.6%)	12,233 (13.5%)	<0.001
Back pain	1216 (23.4%)	20,396 (22.5%)	0.161
Osteoarthritis	392 (7.5%)	9939 (11.0%)	<0.001
Chronic bronchitis/COPD	210 (4.0%)	6679 (7.4%)	<0.001
Cancer	167 (3.2%)	7864 (8.7%)	<0.001

Proportions of patients are given in % unless otherwise indicated. SD: standard deviation.

**Table 2:** Association between Silexan prescription and the probability of a repeated sleep disorder diagnosis within a period of 15–365 days after the index date (multivariable logistic regression).

Variable	Odds Ratio (95% CI) *	*p*-Value
Silexan versus Z-Drugs	0.56 (0.51–0.60)	<0.001
Male vs. female	1.01 (0.98–1.04)	0.659
Age: 18–30 years	Reference	
Age: 31–40 years	1.25 (1.15–1.36)	<0.001
Age: 41–50 years	1.43 (1.32–1.54)	<0.001
Age: 51–60 years	1.64 (1.52–1.76)	<0.001
Age: 61–70 years	1.93 (1.79–2.08)	<0.001
Age: 70+ years	2.49 (2.32–2.68)	<0.001
Private health insurance vs. statutory insurance	1.23 (1.17–1.29)	<0.001
Depression	1.35 (1.30–1.40)	<0.001
Anxiety disorder	1.19 (1.12–1.26)	<0.001
Reaction to severe stress and adjustment disorder	1.06 (1.01–1.12)	0.030
Diabetes	1.33 (1.28–1.39)	<0.001
Back pain	1.14 (1.10–1.18)	<0.001
Osteoarthritis	1.24 (1.19–1.30)	<0.001
Chronic bronchitis/COPD	1.37 (1.30–1.44)	<0.001
Cancer	1.13 (1.08–1.19)	<0.001

* adjusted for sex, insurance status, and co-diagnoses.

**Table 3:** Association between Silexan prescription and the probability of a repeat sleep disorder diagnosis within a period of 15–365 days after the index date, stratified by age group [Silexan versus Z-Drugs] (multivariable logistic regression).

Age Stratification	Odds Ratio (95% CI) *	*p*-Value
Age: 16–30 years	0.56 (0.46–0.69)	<0.001
Age: 31–40 years	0.65 (0.52–0.82)	<0.001
Age: 41–50 years	0.57 (0.47–0.69)	<0.001
Age: 51–60 years	0.53 (0.44–0.63)	<0.001
Age: 61–70 years	0.48 (0.38–0.59)	<0.001
Age: 70+ years	0.58 (0.50–0.68)	<0.001

* adjusted for sex, insurance status, and co-diagnoses.

## Data Availability

The data that support the findings of this study are available on request from the corresponding author on reasonable request.
